# Recent Advances about the Applications of Click Reaction in Chemical Proteomics

**DOI:** 10.3390/molecules26175368

**Published:** 2021-09-03

**Authors:** Tingting Yao, Xiaowei Xu, Rong Huang

**Affiliations:** 1 School of Pharmaceutical Sciences, South-Central University for Nationalities, Wuhan 430074, China; 201721151006@scuec.edu.cn; 2 State Key Laboratory of Natural Medicines, Key Lab of Drug Metabolism and Pharmacokinetics, China Pharmaceutical University, Nanjing 210009, China

**Keywords:** click reaction, chemical proteomics, activity-based protein profiling, hybrid monolithic column

## Abstract

Despite significant advances in biological and analytical approaches, a comprehensive portrait of the proteome and its dynamic interactions and modifications remains a challenging goal. Chemical proteomics is a growing area of chemical biology that seeks to design small molecule probes to elucidate protein composition, distribution, and relevant physiological and pharmacological functions. Click chemistry focuses on the development of new combinatorial chemical methods for carbon heteroatom bond (C-X-C) synthesis, which have been utilized extensively in the field of chemical proteomics. Click reactions have various advantages including high yield, harmless by-products, and simple reaction conditions, upon which the molecular diversity can be easily and effectively obtained. This paper reviews the application of click chemistry in proteomics from four aspects: (1) activity-based protein profiling, (2) enzyme-inhibitors screening, (3) protein labeling and modifications, and (4) hybrid monolithic column in proteomic analysis.

## 1. Introduction

Proteomics is a kind of omics which studies the protein composition, distribution and changing rules in cells, tissues or organisms. Essentially, it refers to the macroscale study of protein characteristics, including protein expression level, post-translational modification, small molecule–protein interaction and so on [1]. Research on the proteome cannot only provide the material foundation for the law of the activities of life, but also provides a theoretical foundation and solutions to elucidate and conquer numerous types of mechanisms of illness [2]. Traditional proteomic methods and analytical approaches have not been able to elucidate the complete network of interactions and modifications that proteins may undergo, nor do they evaluate protein activity or functional state in native environments. Therefore, those challenges emerged have sparked shared interests between chemists and biologists. To conquer these, a myriad of chemical proteomics methods have been developed, among which click chemistry can overcome the limitations of the biological proteome methods, identifying the binding targets in cells and tissues.

**Figure 1:**
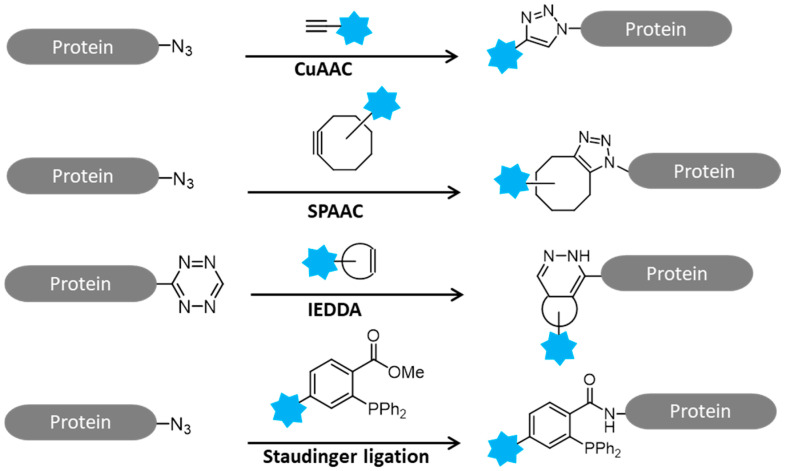
Schematic reactions of click chemistry.

Click chemistry was first put forward by K B Sharpless in 2001 [3] which provides a quick and reliable synthesis method for different molecules to offer a range of reactivities, orthogonality and utility in various applications. Click chemistry is characterized by good chemical selectivity, favorable solvent compatibility, diverse modularization, minimum synthesis requirements and high yield, upon which it considerably reduces the effect of sensor incorporation on protein activity and reveals the structure and functionality of proteins. Click reactions commonly comprise of copper (I)-catalyzed azide-alkyne cycloaddition (CuAAC), strain-promoted azide-alkyne cycloaddition (SPAAC), and inverse-electron-demand Diels–Alder (IEDDA) reaction and Staudinger ligation (Figure 1) [4]. In particular, the Cu(I)-catalyzed version of the Huisgen 1,3-dipolar cycloaddition reaction between azides and terminal alkynes (CuAAC), is the best-known click reaction so far, and has recently emerged to become one of the most powerful tools in chemical biology and proteomic applications [5]. In this paper, the applications of click chemistry in chemical proteomics are summarized and highlighted from four aspects: (1) activity-based protein profiling, (2) enzyme-inhibitors screening (3) protein labeling and modifications and (4) hybrid monolithic column in proteomic analysis.

## 2. Activity-Based Protein Profiling

**Figure 2:**
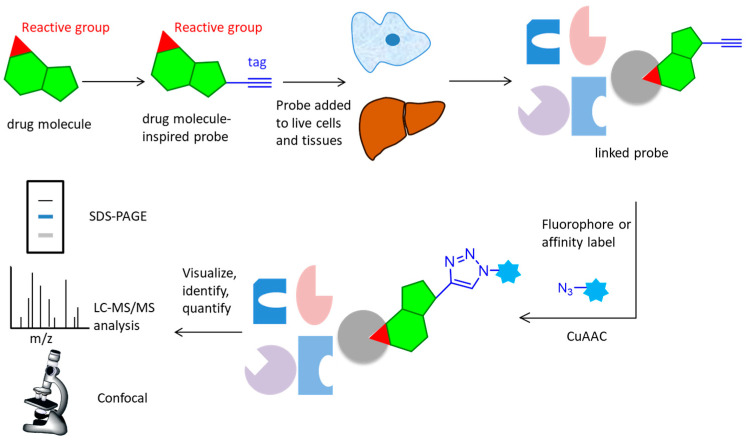
Overall workflow of the activity-based protein profiling approach. A probe with an active group and an alkynyl tag reacts with the active site of target protein in live cells and tissues, then undergoes CuAAC reaction with an azide containing fluorophore or affinity label. After lysis and separation, the probe is fluorescently scanned in SDS-PAGE display, LC-MS/MS analysis, or confocal imaging for identification or quantification.

Over the past few years, activity-based protein profiling (ABPP) has become a strong method of chemical proteomics for analyzing proteins’ functional states within a complex proteome [6,7]. ABPP strategies usually use activity-based probes (ABPs), that are designed to be recognized by the target protein and react with residues from the active site, which can efficiently enrich and identify of low-abundance and low-affinity probe-interacting proteins [8]. Many ABPs have been developed for many classes of enzymes, including serine hydrolases [9,10], cysteine proteases [11], metallohydrolases, phosphatases, deubiquinating enzymes [12,13], kinases [14,15], various oxidoreductases, and others. Although ABPs often utilize reporter groups for the direct enrichment or visualization of labeled proteins, avoiding the need for additional conjugation steps, such bulky groups can hamper cellular uptake and tissue distribution, potentially limiting their application in living systems. To improve these problems, compound-centric chemical proteomics approach on the basis of click reactions were used to enrich and identify enzyme targets, which allow for the incorporation of chemical groups with highly selective reactivity into small molecules, or protein modifications without perturbing their biological function, enabling the selective installation of an analysis tag for downstream investigations (Figure 2). The probe is designed based on the structure of the active molecule, then added to live cells or tissues. It reacts covalently (via an electrophilic trap or a photo-crosslinking group) or non-covalently with the target protein. The lysed samples subject to CuAAC reaction attach a fluorophore, affinity label, or a combination of these elements. Marked proteins are subsequently visualized, identified or quantified using a variety of techniques, such as SDS-PAGE, LC-MS/MS analysis or confocal imaging [8]. The CuAAC-enabled ABPs were developed for protein arginine deiminases [16]; ubiquitin mechanisms [12,17], cytochrome P450 enzymes [18], glycosidases [19], and kinase [20,21].

Clickable probes based on light affinity can be the most common chemical proteomics tool for capturing and identifying non-covalent targets for small bioactive molecules [22]. These probes include the synthesis and installation of a click group (azide or alkine) and photoaffinity groups (such as diazirines, benzophenone) to cause as little disruption as possible to the biological activity of the compounds, which sometimes necessitates a thorough SAR investigation and even new synthetic channels [23,24,25]. Cell permeable sensors with both clickable group and light affinity have been developed to characterize a variety of clinically approved medications and inhibitors, including kinase inhibitors [26], γ-secretory enzyme inhibitors [27], β-secretor inhibitors [28], antibiotics [29], NSAIDs [30], epigenetic regulatory compounds [31], natural products [32], and also protein interactions with lipids [33] and sterol [34]. Considering that metabolites may participate in functional sites of proteins, the metabolite-derived click probes can be used as a valuable analytical tool to plot drug interactions in the proteomes, and even as a tool for uncovering functional regulators of metabolite binding proteins [33].

**Figure 3:**
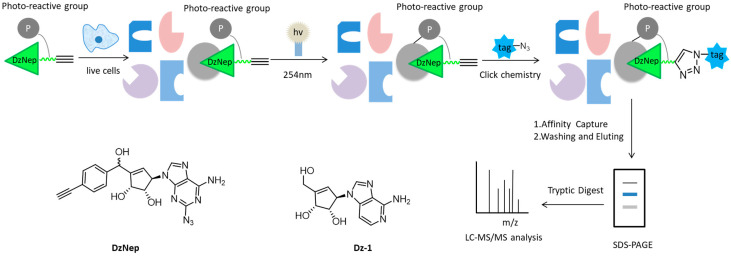
Overall workflow of the cell-based proteome profiling approach followed by large-scale pull-down/LC-MS/MS for identification of potential cellular targets of DzNep using affinity-based probe DZ-1.

As a carbocyclic analog of adenosine, 3-Deazaneplanocin A (DzNep) can inhibit the activity of histone lysin methyltransferase, which has aroused great interest in epigenetic research over the past few years [35,36,37]. However, the molecular mechanism and extracellular targets of DzNep have not been fully understood. Yang et al. developed some small-molecular probes derived from DzNep that are permeable to the cells, but the bulky modification groups in the probes usually disrupt the interactions between proteins and drugs, so it is still challenging to efficiently capture cell targets in situ interactions [26,38,39,40]. Therewith, Tam and colleagues designed a novel “clickable” affinity-based probe DZ-1, with minimal structural modification from DzNep (Figure 3). DZ-1 possessed comparative anti-apoptotic activity as DzNep in MCF-7 mammalian cells. In situ proteome profiling of DZ-1 was successfully carried out on the basis of pull-down LC-MS/MS analysis. Finally, some highly enriched proteins were identified as potential cellular protein targets of DzNep [41].

**Figure 4:**
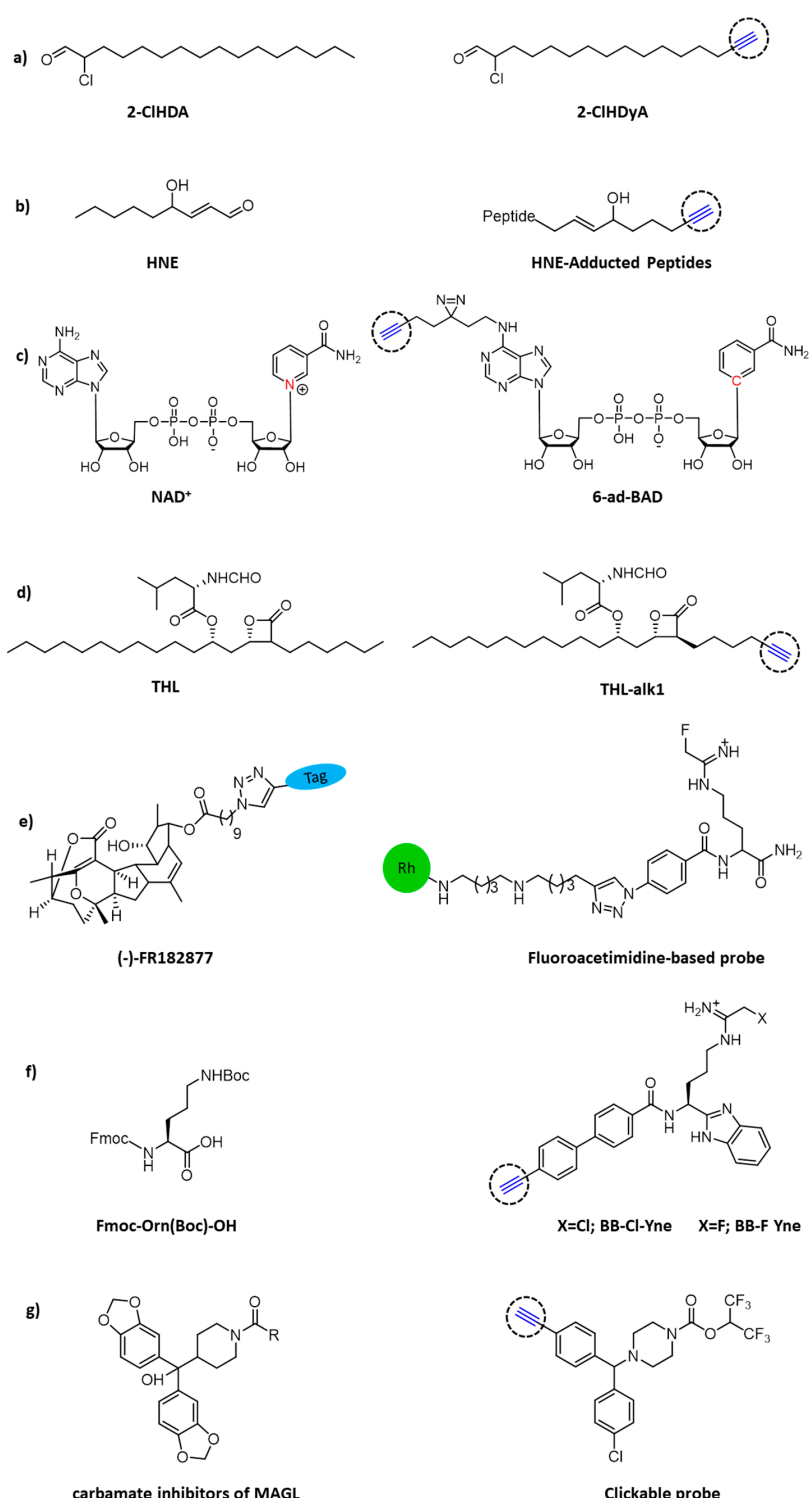
Chemical structures of clickable probes in ABPP for identifying protein targets of: (**a**) 2-ClHDA, (**b**) HNE, (**c**) NAD^+^, (**d**) THL, (**e**) natural product (-)–FR182877, (**f**) Cl- and F-amidine, and (**g**) carbamates.

Inflammation-related processes are pivotal factors contributing to sepsis-associated cardiac dysfunction. Cardiac neutrophil infiltration and subsequent release of myeloperoxidase leads to the formation of the oxidant hypochlorous acid (HOCl) that is able to chemically modify plasmalogens into 2-chlorohexadecanal (2-ClHDA). To elucidate this metabolic process and characterize protein targets for 2-ClHDA, a clickable alkynyl analog, 2-chlorohexadec-15-in-1-al (2-ClHDyA), was used by Prasch and colleagues to identify its protein targets (Figure 4a). Through CuAAC reaction of 5-tetramethylrhodamine azide (N3-TAMRA) and two-dimensional gel electrophoresis, they were able to pinpoint 51 proteins which form adducts with 2-ClhdYA. Genetic ontology enrichment analysis showed that heat shock and chaperone proteins, energetic metabolism and cytoskeleton proteins were the key targets for HOCl modified lipids in the heart of mice with endotoxemia [42].

Diffusible electrophilic α, β-unsaturated aldehydes, such as 4-hydroxynonenal (HNE), are primary targets of free radical damage during oxidative stress. Some studies have shown that HNE or other electrophilic agents can modify IκB kinase (IKK) [43], tubulin isomer [44], and Keap1 [45,46], leading to the loss of protein function and disturbance of cell signal transmission. In order to fully understand the effect of oxidative stress on cellular signaling transduction and disease pathology, it is necessary to analyze HNE modified proteins in vivo. Vila and colleagues explored Staudinger’s ligation and CuAAC to selectively label proteins with HNE in colon cancer cells, and subsequently pull-down by biotin–streptavidin interaction for LC-MS/MS analysis (Figure 4b). The results showed that both strategies produced effective biotinylation of HNE-conjugated protein, while click chemistry was proven to be superior for recovering proteins [47].

Nicotinamide adenine dinucleotide (NAD^+^), known as oxidoreductase coenzyme, is also a multifunctional substrate of many post-translational modification enzymes, such as poly-ADP−ribose polymerases (PARP) and sirtuins [48]. The recent studies of noncanonical NAD-binding proteins suggest that powerful chemical tools for profiling the NAD interactome are quite necessary. Šileikytė and colleagues developed a NAD^+^/NADH probe, 6-ad-BAD, with two reactive sites for both click reaction and light crosslinking. Moreover, the nicotinamide linked to ribose was replaced by a benzamide adenine dinucleotide (BAD) to avoid enzyme digestion (Figure 4c). Results showed that 6-ad-BAD could label PARP effectively in a UV dependent manner. Then, the chemical proteomics of 6-ad-BAD was evaluated in HEK 293T cell lysate through biotinylated enrichment and 24 unknown NAD or related nucleotide binding proteins were identified. This clickable probe will be useful in future chemical proteomics studies for profiling the NAD^+^ interactome across different tissues as well as in disease contexts [49].

Tetrahydrolipstatin (THL, also known as Orlistat) is an FDA approved anti-obesity drug with potential bactericidal activity. To explore the enzymatic targets of this β-lactam ring in a complex bacterial proteome, Ravindran and Wenk designed a functional THL–alkyne analog (Figure 4d) to quantify the lipid esterase activity and enriched the target proteins in *Mycobacterium bovis* BCG at different physiologic states [50].

Adam and colleagues used click chemistry as a handy binding method to synthesize both rhodamine-, and rhodamine and biotin-tagged (trifunctional) sensors from the natural product (-)–FR182877 [51]. Using this sensor, the researchers identified carboxyesterase-1 as the protein target in the heart of the mouse (Figure 4e). In the same way, Thompson et al. used click chemistry to covalently label rhodamine with inhibitors for arginine deiminase 4 (PAD4), an enzyme controlled by calcium [52].

Citrullination is the post-translational hydrolysis of peptidyl-arginine to form peptidyl-citrulline [53,54,55], which is a reaction also catalyzed by PADs. Abnormal increase of citrullinated protein is associated with autoimmune illnesses and cancers [53,56]. Cl-amidine and F-amidine were reported to permanently inhibit PADs by covalently altering an active cysteine site [57,58,59]. Herein, Nemmara and colleagues developed cell permeable and “clickable” probes (BB-Cl-Yne and BB-F-Yne) for covalent labeling of the PADs both in vitro and in cell-based systems [9]. These sensors covalently alter the conserved cysteine residues in all PAD isozymes and serve as the base for azide-alkyne cycloaddition, and, subsequently, CuAAC [60] with either TAMRA-N_3_ or biotin-N_3_. It is worth noting that such compounds may be used in several forms, including the off-target recognition of the parent compounds and ABPPs on the target binding tests, to demonstrate PAD inhibitor efficacy.

Chang and colleagues used a combination of competitive and click chemistry ABPP (Figure 4g) to study a variety of proteomic reactions to activate carbamates in vitro and in vivo. They identified several carbamates derivatives, among them, *O*-aryl and *O*-hexafluoroisopropyl (HFIP) carbamates could react selectively with serum hydrolases in vivo. They used the proteomic specificity of carbamate HFIP to plot in situ images of monoacylglycerol lipase endocannabinoid hydrolases and *α-β* hydrolase-6. They proved that carbamates are the preferred reaction group of serine hydrolase, which can adapt to different structural modifications and produce inhibitors with special potency and selectivity in the mammalian proteome [61].

Designed to identify chemical probes for functionally related protein families that can be used in complex natural environments, ABPP is an area that benefits from click chemistry. Due to the modular and efficient properties of the click reaction, the synthesis of probe libraries has been greatly simplified. However, there is still lack of a universal chemical probe or small molecule ligand for all target proteins, and the real-time dynamic imaging of a specific protein of interest in a live organism remains highly challenging. Due to the diversity of biological applications, there is no standardized protocol covering the different aspects of probe preparation, click reaction conditions, or analysis. This results in various methods that may seem overwhelming to the newcomer. Click reactions can be readily integrated to both conventional biological techniques such as gel-based fluorescent-labeling, biotin-based pull-down assay, and many of the upcoming high-throughput bioassays and characterization techniques such as microarray, LC-MS/MS, etc. Furthermore, click modules enable the enrichment of protein targets, yet their presence can sometimes perturb molecular interactions and biological activity. Recent advances in ‘‘label-free’’ proteomic methods, such as thermal proteome profiling or drug affinity responsive target stability, represent complementary strategies to plot small molecule–protein interactions, bypassing the requirement of enrichment handles.

## 3. Enzyme-Inhibitors Screening

Target-guided synthesis (TGS) is mainly divided into dynamic combinatorial chemistry and kinetically controlled TGS. Tethering and in situ click chemistry are representative strategies, respectively. The former method is based on thiol-disulfide exchange, in which free sulfhydryl groups on the protein surface react with small fragments containing disulfide bonds to form disulfide bonds [62], the latter uses irreversible click reactions to synthesize two reaction building blocks into a potentially inhibiting compound [63].

**Figure 5:**
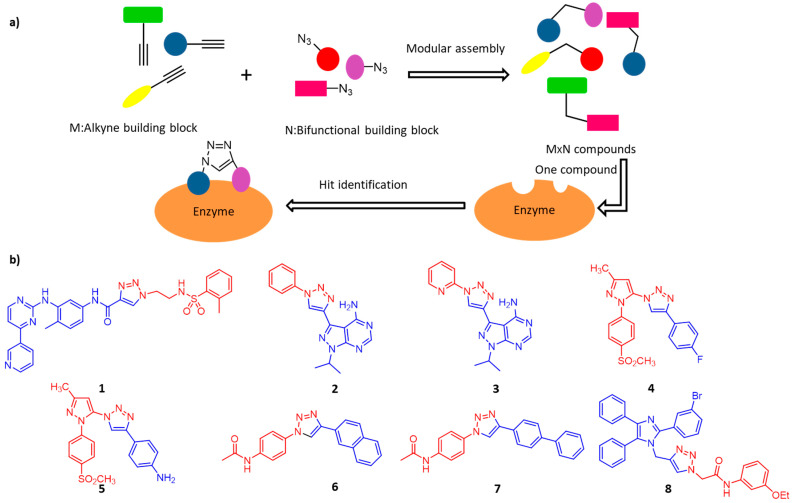
(**a**) Illustration of in situ click chemistry contributes to rapid assembly of potential bidentate inhibitors (M × N compounds) from N number of alkynes and M number of azides. (**b**) Potent and selective click-inhibitors of **1**: Abl kinase, **2** and **3**: PfPK7, **4** and **5**: COX-2, **6** and **7**: OGT, and **8**: α-Glucosidase.

Traditional drug development usually depends on pharmaceutical chemistry, whether in the very beginning of drug discovery or the subsequent stages of drug optimization. Many enzymes have multiple binding domains, and apart from the active center, the allosteric binding sites mainly confer selectivity and potency [64]. In this case, click chemistry is considered to be a convenient method for assembling fragment-based inhibitors because of its highly modular and efficient reaction characteristics. As illustrated in Figure 5a, the combinations of M+N fragments result in potential bidentate inhibitor library (M×N compounds) for high-throughput screening [65].

### 3.1. Protein Kinases

Protein kinases catalyze the phosphorylation of serine, threonine, tyrosine and histidine residues of proteins. Aberrant kinases expressions are involved in numerous illnesses, including inflammation and cancers [66]. Kalesh and colleagues recently used click chemistry to produce 344 Abelson tyrosine kinase (Abl) inhibitors [67]. Later inhibition screening assays showed that Abl kinase had a preference for short chain azide scaffolds, then 11 lead compounds with moderate potency were found. Among them, compound **1** is the most potent hit during screening with IC_50_ = 700 nM (Figure 5b). Similarly, Klein and colleagues used this strategy to produce *Plasmodium falciparumprotein* kinase 7 (PfPK7) inhibitors [68]. The researchers used alkyne/azide-derivatized purine analogs to click on various aromatic azides/alkynes, and subsequent inhibition screening assays resulted in two potent PfPK7 inhibitors (compound **2** and **3,** Figure 5b) with IC_50_ at 10–20 μM.

### 3.2. Cyclooxygenase-2

Cyclooxygenase (COX) catalyzes the transformation of arachidonic acid into prostaglandins, which plays an important role in human physiology and pathological conditions [69]. Among the three subtypes of COX, COX-2 is considered to be closely related to various pathological processes, so the development of selective COX-2 inhibitors is a major focus of pharmaceutical research. Bhardwaj and colleagues demonstrated the use of the COX-2 binding site as a reaction vessel to produce its own potent and selective inhibitors. They have designed and synthesized a series of pyrazole-based azide building blocks and a series of corresponding triazole-containing biheterocyclic compounds through the in situ click chemistry method, and screened out compounds **4** and **5**, (Figure 5b), which are highly effective inhibitors of COX-2 [70].

### 3.3. O-GlcNAC Transferase

*O*-GlcNAC transferase (OGT) is a critical enzyme involved in the dynamic *O*-GlcNAcylation of nucleoproteins and cytoplasmic proteins. The discovery of cell permeability OGT inhibitors is of great significance to elucidate the function and regulatory mechanism of *O*-GlcNAcylation [71]. Wang et al. combined the advantages of tethering and in situ click chemistry to find OGT inhibitors. They reported two cell permeable OGT inhibitors (compounds **6** and **7**, Figure 5b), both of which significantly inhibited intracellular *O*-GlcNAcylation without side-effects on cell viability. Unusual non-competitive inhibition of OGT was helpful to find new inhibitors and explore the regulatory mechanism of OGT [72].

### 3.4. α-Glucosidases

The family of enzymes α-Glucosidases play an important role in carbohydrate digestion in vivo [73]. Inhibition of α-glucosidase activity could reduce the level of plasma glucose after surgery, therefore it has been considered as an important target for the treatment of type II diabetes mellitus [74,75]. Wang and colleagues synthesized a series of 2,4,5-triarylimidazole-1,2,3-triazole derivatives using CuAAC and evaluated their inhibitory effects on α-glucosidase. Among them, a new type of structural α-glucosidase inhibitor (compound **8**, Figure 5b) was identified, which can be used as a lead compound for further development of α-glucosidase inhibitors [76].

The development of enzyme inhibitors is another important area where click chemistry plays a positive role. It is considered to be a convenient fragment-based inhibitor assembly strategy, in which a large number of potential bipedal inhibitors are generated with minimum synthetic effort. Ingenious strategies such as in situ click chemistry have so far shed some light on new ways of producing potent inhibitors against certain enzymes. However, this strategy is still in its infancy, requires a large amount of protein, and the amplification effect is relatively poor except in some highly optimized conditions.

## 4. Protein Labeling and Modifications

Protein labeling and modification are widely used in industry, agriculture, and medicine, and have high research value. Protein labeling refers to the process of covalently linking enzyme, fluorescein, biotin and other markers to antibodies or other proteins, and specifically reacting with the detected products to form multiple complexes [77]. Protein engineering is based on the relationship between the structure of protein molecules and their biological functions. Through chemical, physical and molecular biological methods, gene modification or gene synthesis is carried out to modify the existing protein, or to produce a new protein to meet the needs of human production and life [78]. Next, we will summarize the applications of click chemistry in the labeling of new proteins, post-translational modification of proteins, and protein engineering.

### 4.1. Labeling of New Proteins

**Figure 6:**
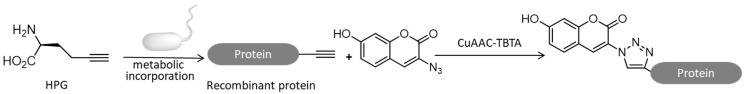
TBTA assisted CuAAC labeling of newly synthesized proteins in bacterial cells. Met analogs containing terminal alkyne were metabolized and incorporated into target proteins in bacterial cells, and then TBTA assisted CuAAC to react with azide modified coumarin fluorophores.

Although CuAAC has a variety of ligands, these reactions are mainly limited to cell surface markers [79]. The direct labeling of intracellular biomolecules remains to be explored to a great extent. Beatty and colleagues reported for the first time the utility of the CuAAC reaction for labeling newly synthesized proteins in bacteria (Figure 6). *Escherichia coli* cells were treated with 19 kinds of natural amino acids and alkyne functionalized unnatural amino acid, homopropargylglycine (HPG), to prepare recombinant protein Bastar with alkyne group. Previous work has demonstrated that alkynyl amino acids can be incorporated easily into recombinant proteins in a residue-specific manner. HPG serves effectively as a methionine (Met) surrogate even without modification of the translational machinery of the host. Then, the bacterial cells were treated with tris (benzyltriazolylmethyl) amine (TBTA) and azide functionalized coumarin at 4 °C for 14 h for CuAAC reaction. After a large amount of washing, cells were stimulated at 395 nm, and the fluorescent emission was monitored. Only when HPG, azido-coumarin and Cu (I) TBTA were co-incubated the cells, the fluorescence intensity increased significantly. Confocal fluorescence microscopy and polyacrylamide gel electrophoresis confirmed the successful labeling of the Bastar protein in *E. coli* [80].

### 4.2. Protein Post-Translational Modifications

Protein post-translational modifications (PTMs) refer to a covalent process during or after protein translation, that is, to change the properties of proteins by adding modification groups to one or several amino acid residues or cutting off groups by protein hydrolysis. More than 300 different PTMs have been found, including phosphorylation, glycosylation, acetylation, ubiquitination, carboxylation and disulfide pairing [81].

**Figure 7:**
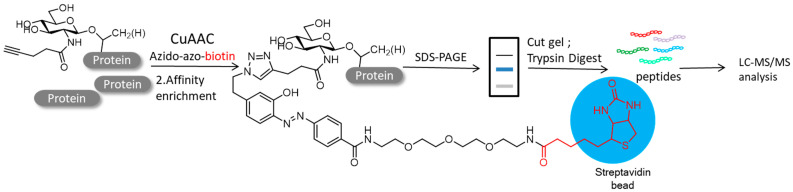
GlcNAlk is metabolized and integrated into NIH-3T3 cells. After lysis, the protein was treated with azido-azo-biotin for CuAAC. GlcNAlk modified proteins were isolated by affinity enrichment based on streptavidin beads.

Among the different PTMs, protein glycosylation regulates important mechanisms associated with cellular communication, which plays a major role in immune reactions, inflammation and cancerous metastases [5]. Some studies have reported a mutant galacytosyltranferase which can transfer an N-azidoacetylgalactosamine (GalNAz) residue onto O-GlcNAc modified proteins, therefore the bio-orthogonal reaction provided an elite chemical method for glycomics studies [82,83]. Zaro and colleagues exploited click reactions to isolate and identify glycosylated proteins. Specifically, they demonstrated the metabolic intake of N-acetylglucosamine analog GlcNAlk on *O*-GlcNAcylated proteins in NIH-3T3 cells. After lysing in SDS, the azido-azo-biotin was applied to the soluble proteins, followed by enrichment with streptavidin beads. The biotinylated proteins were then released upon sodium dithionite treatment and separated with SDS-PAGE (Figure 7). Ultimately, the LC-MS resulted in the identification of 374 proteins modified by GlcNAlk [84].

**Figure 8:**
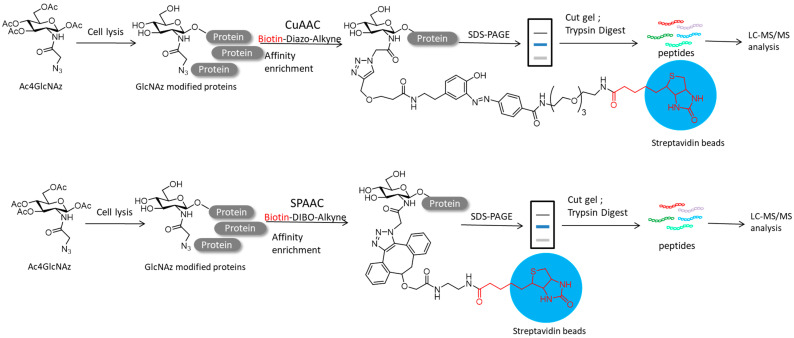
*O*-GlcNAcylated protein was metabolized with azide GlcNAc analog (Ac4GlcNAz) using Biotin-Diazo-Alkyne (CuAAC)/Biotin-DIBO-alkyne-probe (SPAAC).

Li and colleagues developed a gel mass spectrometry method to identify *O*-GlcNAC modified proteins using peracetylated N-azidoacetylglucosamine (Ac4-GlcNAC) in A549 cells. After conjugating with click chemistry (CuAAC and SPAAC) in vitro and streptavidin resin enrichment, the *O*-GlcNAc modified proteins were isolated by SDS-PAGE and identified by mass spectroscopy (Figure 8). Analysis of the proteomic data indicated that 229 suspected modified *O*-GlcNAc proteins were identified with a conjugate sample of Biotin-Diazo-Alkyne and 188 proteins with a conjugate sample of Biotin-DIBO-Alkyne, of which 114 overlapped. This method combined with metabolic markers, click chemistry, affinity enhancement, SDS-PAGE separation and mass spectrometry will adapt to suit for other PTMs of proteomics [85].

**Figure 9:**
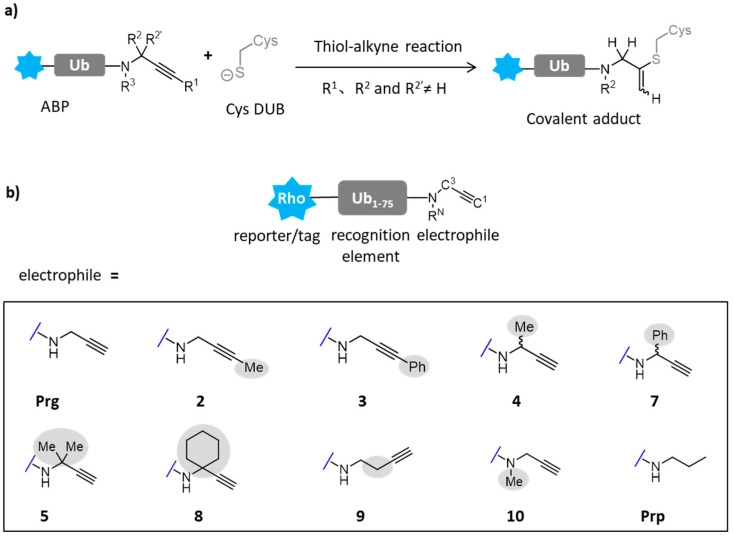
(**a**) Mechanism of activity-based probe targeting cysteine DUBs via thiol alkynyl click reaction. (**b**) A group of substituted alkynes in active probes (ABPs) targeting cysteine DUBs. Synthetic ubiquitin lacking glycine residues at the C-terminal (Ub_1−75_) was modified with fluorescent rhodamine group at the N-terminal as reporter, and propargylamide (Prg) or propargylamide derivative **2**–**10** at the C-terminal as cysteine thiol-reactive electrophilic. Propylamide (Prp) was a non-covalent control.

As a common post-translational modification, ubiquitination can regulate a variety of protein substrates in different cellular pathways [86]. Meanwhile, deubiquitinating enzymes (DUBs) realize the process of deubiquitination by cutting the isopeptide bond between the C-terminal of ubiquitin and the lysine residue of the target protein, or by polymerizing the isopeptide bond between the distal and proximal ubiquitin of the ubiquitin chain. The unactivated terminal alkynyl group in active probes is considered as a standard reaction site for labeling cysteine deubiquitinating enzymes (Figure 9a). These probes are widely used to monitor the activity of DUBs in infection, disease and treatment, or to discover new DUBs and their active cysteine. Mons and colleagues have designed a series of probes to study the reactivity of terminal alkynyl groups towards DUBs (Figure 9b). The core of the probes is rhodamine linked to the total synthesized ubiquitin_1–75_(Ub_1–75_). Different reaction groups were attached to the carbon end of ubiquitin, in which **2** and **3** are replaced by methyl and phenyl, respectively. In order to study the importance of terminal protons, the substitution at C3 makes the volume of the probe larger (**4**, **7**). Propionamide served as a negative control because it had no reactive group. The possibility of thiol alkynyl click reaction targeting deubiquitinating enzymes in cells was studied. Moreover, the target range of propargyl derivatives targeting cysteine was extended from terminal alkynyl to internal substituted alkynyl [87].

### 4.3. Protein Engineering

Natural microbial rhodopsin is a seven-fold transmembrane protein. As a kind of photosensitive ion channel or ion pump, it has been widely used in optogenetic modulation [88]. The genetically encoded voltage indicators of rhodopsin were modified to be genetically encoded membrane potential probes by electrochromic detection of membrane potential. On the basis of Förster resonance energy transfer, a rhodopsin type membrane potential probe was developed, which can realize an all-optical study of electrophysiology of cultured neurons. Peng’s research group designed a series of fluorescent membrane potential probes hybrid voltage indicator (HVI) with high sensitivity and signal-to-noise ratio. They used the enzyme mediated probe incorporation method to specifically bind the trans-cyclooctene part to the mutants of Acetabularia acetabulum rhodopsin II (Ace2), which was subsequently derivatized with tetrazine-conjugated organic fluorophores via the inverse-electron-demand Diels–Alder cycloaddition (IEDDA) reaction. The resulting HVI had a dye protein structure and exhibited a strong electrochromic effect, which can be used for an all-optical electrophysiological study of cultured neurons [89].

The application of click chemistry in protein labeling and modification has greatly promoted the development of chemical proteomics. However, it is important to note that most clinically approved drugs target membrane receptors, but relatively few chemical probes have been developed for this broad class of proteins. The click reaction for new protein labeling is operationally similar to conventional pulse-labeling with ^35^S-methionine but avoids the technical challenges of high-resolution autoradiography. That being said, further work for the protein labeling with alkynyl amino acid side chains in mammalian cells is needed, and click chemistry has been used in only a few types of PTMs. Therefore, determining the exact location and exact structure of modifications on proteins remains challenging.

## 5. Hybrid Monolithic Column in Proteomic Analysis

Since the end of 1980s, Hjertén and Svec first used monolithic columns as steady-state phases for rapid separation of small and macro molecules [90,91]. Therefore, different monolithic columns containing organic polymer, inorganic silicon or a hybrid matrix of organic silicon were reported [92,93]. At present, organic monolithic columns and inorganic silicon monolithic columns have been largely utilized in the separation field [94,95]. Monolithic columns of organic polymer normally contain polyacrylamide, polyacrylate, polymethylmethacrylate and polystyrene, which have good acid and alkali resistance, simple preparation and easy surface modification [96]. However, they will dilate in certain organic solvents, potentially altering the pore structure of polymer matrix, thereby reducing the mechanical stability and service lifetime. On the contrary, inorganic silica gel has good resistance to organic solvents and high mechanical stability, but its surface functionalization requires tedious operation process and more time, which limits the application of monolithic silica column [97,98]. The organic-inorganic hybrid monolithic column combines their respective advantages with large specific surface area, high performance, and convenient preparation. The hybrid monolithic column prepared or modified by thiol-ene click reaction can be used to separate and purify proteins.

Liu and colleagues developed hybrid monolithic silanes by co-condensing tetramethoxysilane and vinyltrimethoxysilane and studied the change of vinyl content of hybrid monolithic silanes treated with vinyldimethylexysilane. In addition, through the thiol-ene click reaction of vinyl with 1-octadecanethiol, sodium 3-mercapto-1-propanesulfonate and 2,2′-(ethylenedioxy)diethanethiol/vinylphosphonic acid, the surface properties of the whole matrix can be easily adjusted, which can be used as the analytical column in LC-MS/MS for separating and analyzing the tryptic digest of the HeLa cells [99].

**Figure 10:**
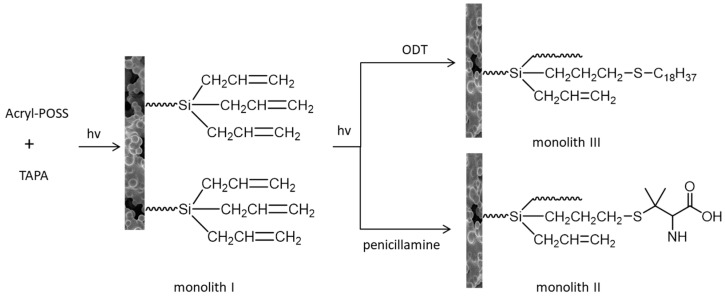
Preparation of monolithic column by two-step photo-initiation reaction.

Polyhedral oligomer silsesquioxane (POSS) is a hybrid material with cage-like structure and nanoscale size [100,101]. The inherent properties of POSS, such as good resistance to temperature/oxidation and an excellent tolerance to pH, make it appropriate to prepare a new type of monolith column [102,103]. Ma and colleagues reported a pure POSS-based hybrid monolithic column (Figure 10), which was prepared by self-polymerization of acrylate (monolith I) with acrylopropyl polyoctahedral silsesquioxane (acryl-POSS) as crosslinking agent and 3-(triallyl silyl) propyl acrylate (TAPA) as a monomer. As a result, monolithic column I can be further modified by thiol-ene click reaction with penicillamine (monolithic II) and 1-octadecanethiol (monolithic III). The three monolithic columns were characterized and then applied to the separation of various small molecules and the determination of trypsin digestion solution of HeLa cells by reciprocal liquid chromatography [104].

Wu and colleagues constructed a POSS-based hybrid monolithic column using methacryl substituted POSS and N-(2-(methacryloyloxy)ethyl)-dimethyloctadecyl ammonium bromide [105]. The column showed excellent mechanical stability and better chromatographic performance than conventional alkoxysilane-based monolithic columns without tailing spikes, which could successfully separate proteins from tryptic digests of bovine serum albumin [106,107]. In addition, a new POSS-VBI-Cys column, developed by Han et al., demonstrated good separation selectivity for glycoprotein and non-glycoprotein. It is worthy to exploit its performances for separation of intact proteins and in-depth proteome applications [108].

## 6. Considerations and Looking Forward

Looking back at the past twenty years, the introduction of chemical strategies to parse and enrich subsets of the ‘‘functional’’ proteome has empowered mass spectrometry (MS)-based methods to delve more deeply and precisely into the biochemical state of cells and its perturbations by small molecules. The emerging click chemistry provides a powerful tool for the proteomics research, fundamentally promoting the development of chemical biology and molecular biology. This paper mainly highlights the applications of click chemistry in activity-based protein profiling, enzyme-inhibitors screening, protein labeling and PTMs, and hybrid monolithic column for protein purification. ABPP and chemical proteomics are designed to identify the chemical probes of allied functional protein families, which can be used in a complex natural environment. Because of the modularization as well as high efficiency of click reaction, synthesizing a diverse compound library for high-throughput screening of enzyme inhibitors is becoming much easier. In spite of these attributes, the efficiency of click reaction needs to be further improved. Some PTMs are large and heterogeneous, making their recognition by proteomics software challenging, thus PTM-selective enrichment is necessary prior to MS analysis. However, we should be clear that the application areas of click chemistry in PTMs are limited. The development of specific sensors for all kinds of protein post-translational modifications remains to be a major research focus in the future. Furthermore, optimization for the non-toxic click reaction conditions, and strategy development with superior tissue penetrating ability to conduct sensitive metabolic labeling experiments on living animals are also pointcuts and considerations for future research. We can expect researchers to delve further into the application of click chemistry in the cutting edge of proteomics, contributing significantly towards a better understanding of our life processes.

## Data Availability

Not applicable.
